# Hands-On Defibrillation—The End of “I'm Clear, You're Clear, We're All Clear”?

**DOI:** 10.1161/JAHA.112.005496

**Published:** 2012-10-25

**Authors:** Richard E. Kerber

**Affiliations:** Department of Internal Medicine, University of Iowa College of Medicine, Iowa City, IA

**Keywords:** editorial, cardiopulmonary resuscitation, defibrillation, ventricular fibrillation, electric current, polyethylene glove, hands-on defibrillation

The importance of closed-chest compression in maintaining at least a minimum of myocardial blood flow during cardiac arrest has been increasingly recognized. The adverse effect of interruptions of chest compressions on coronary perfusion pressure is immediate and important; Berg et al showed a 40% decrease in cumulative coronary perfusion after a 13-second pause in chest compression.^1^ The 2010 American Heart Association Guidelines for CPR and Emergency Cardiac Care specifically advise rescuers to minimize interruptions of chest compressions for checking the pulse, analyzing rhythm, or performing other activities throughout the entire resuscitation, particularly in the period immediately before and after a shock is delivered (class IIA, level of evidence B).^2^ In practice, however, an interruption in chest compressions always happens in the period immediately before a defibrillating shock is delivered; to protect the individual performing the chest compressions from inadvertently being incorporated into the current pathway and thereby suffering the possibly lethal passage of electric current, rescuers have long been advised to stop compressions and move away from the patient. The ritual chant “I'm clear, you're clear, we're all clear” serves as a mnemonic for this purpose. Although intended to protect the rescuer from harm, interruption of chest compressions and a fall in myocardial perfusion must result, an unintended and undesirable byproduct.

Is this practice of “clearing” the patient before defibrillation really necessary? In 2008, Lloyd et al^3^ undertook to intentionally put themselves into the current pathway of biphasic shocks administered during elective cardioversion of atrial fibrillation. They wore polyethylene gloves, self-adhesive external electrode pads were used, and the actual current flow through the “rescuers' ” bodies was measured. The results were noteworthy — none of the rescuers felt the shock, and the current flow through their bodies was minimal, less than the leakage current that typically occurs from electric kitchen appliances. In an editorial that accompanied the article by Lloyd et al, this writer wondered if the American Heart Association should revisit its long-standing admonition to “clear” the patient about to receive a defibrillating shock; eliminating this recommendation would advance the goal of minimizing chest compression interruptions during CPR.^4^

The article “Hands-On Defibrillation Has the Potential to Improve the Quality of Cardiopulmonary Resuscitation and Is Safe for Rescuers” in this issue of *JAHA*, by Neumann et al,^5^ continues this discussion. In a porcine model of cardiac arrest, 20 anesthetized swine underwent an initial 7 minutes of electrically induced ventricular fibrillation (VF) followed by CPR (chest compressions and oxygen) beginning after 7 minutes of VF. After 11 minutes of VF, the animals were defibrillated with biphasic shocks delivered through pregelled self-adhesive defibrillation electrodes with nonconductive backing. The swine were divided into 2 groups; “Hands-Off” defibrillation, where the rescuers “cleared” the animal prior to the shock, and “Hands-On” defibrillation, where the rescuers, wearing 2 pairs of polyethylene gloves each, continued chest compressions as the shocks were being delivered. In the “Hands-Off” animals, chest compressions were interrupted for 8.2% of the total CPR time, whereas in the hands-on group, compression interruptions (for rhythm analysis, not for defibrillation) only totalled 0.8% of the total CPR time (*P*=0.0003).

Berg et al^1^ emphasized that following an interruption in chest compressions, coronary perfusion pressure does not immediately return to its preinterruption level, but requires several additional compressions to do so. In the Neumann study, a coronary perfusion pressure (CorPP) “restoration time” was defined as the interval from restarting CPR to the moment when coronary perfusion pressure reached its preinterruption level. If after an interruption, the coronary perfusion pressure was not restored to its preinterruption level, the interval from restarting CPR to the next interruption was measured, and the authors calculated a “CorPP restoration ratio,” the cumulative CorPP restoration time divided by the total CPR time. The CorPP restoration ratio in the hands-on group was only 1.9% of the total CPR time versus 6.3% in the hands-off group (*P*=0.02). Peak lactate concentration was seen 5 minutes after resumption of spontaneous circulation (ROSC) in the hands-on group versus 2 hours after ROSC in the hands-off animals, implying a more intensive reperfusion in the hands-on swine. The rescuers did not feel any electrical stimulus from the shocks, nor were any serious arrhythmias observed on ECG monitoring.

These findings are impressive. However, there was no difference in ROSC between the 2 groups. The differences in CPR quality, which are convincingly demonstrated in this study, are only surrogate end points in the absence of an improvement in ROSC, which is itself a surrogate end point for the ultimate goal, increased survival from cardiac arrest with no neurologic damage. Does this indicate that improvements in CPR quality, much emphasized recently, will not yield improvement in survival? The design of this study, unfortunately, does not afford an answer to this question. The duration of unsupported VF that the experimental protocol mandated was only 7 minutes before chest compressions were begun. Surely this relatively short period of VF accounted for the high ROSC rate in both groups (90% hands-on, 80% hands-off, not significant). If this experiment were to be repeated with a longer duration of untreated VF — perhaps 15 minutes — such a more challenging protocol would have an increased likelihood of showing a ROSC difference in favor of hands-on defibrillation.

Neumann et al^5^ also raise the important point that hands-on defibrillation may make detection of successful resuscitation difficult, not only immediately postdefibrillation but also during sustained chest compressions, which induce motion artifacts, thus hindering rhythm analysis. The authors used marked increases in blood pressure and etCO_2_ during CPR to recognize success; they also pointed out that sophisticated ECG filter software, adaptive noise cancellation techniques, and wavelet-based transformation and shape-based morphology detection are promising technologies to improve rhythm analysis during CPR.^6,7^

The present study of Neumann et al^5^ supports the earlier report of Lloyd and colleagues^3^ in that although the investigators felt the shock-induced muscular contractions of the pigs' bodies, they did not sense any electrical current, nor were arrhythmias provoked in the investigators. However, the safety of the hands-on technique remains a crucial consideration. Petley et al^8^ recently published a detailed discussion of the factors that determine current flow through rescuers in contact with subjects being defibrillated. Rescuers in such situations are working in close proximity to voltages up to 5000 V; voltages this high pose risks to rescuers, patients, and bystanders. The magnitude of the escape current passing through a rescuer is determined by the pathway resistance, which in turn is dependent on glove integrity, rescuer skin moisture content, and the actual pathway taken by the current; transmyocardial pathways may induce fatal arrhythmias, whereas transneural current can cause epilepsy or nerve damage.^8^ The familiar postdefibrillation skin “burns,” outlining the shape of the electrodes used, are the result of transdermal passage of current flow that preferentially follows the edges of the defibrillation electrode.^9,10^ At the 30- to 40-ampere current levels achieved during defibrillation, current can cause direct tissue damage through direct cellular breakdown, tissue necrosis, and burn injury from a heating effect proportional to the square of the current. High-frequency currents can cause electrical stimulation of tissues, inducing ventricular fibrillation. Polyethylene gloves such as worn by Neumann and colleagues^5^ (2 pairs each) may break down at high voltages^11^ and in any case are not standardized by manufacture, content, or insulating effectiveness. Readers interested in this topic should consult the comprehensive and informative discussion of Petley et al.^8^

In sum, the experiment of Neumann et al^5^ has advanced the discussion of hands-on defibrillation from the safety of the technique (the focus of Lloyd et al^3^) to the possible clinical benefits of the technique, which are consonant with the AHA's emphasis on minimizing interruptions of closed-chest compression during CPR. Should the AHA consider a guideline modification, recommending that chest compressions be continued through defibrillation provided that self-adhesive electrodes are used and gloves are worn?^4^ Before such a guideline change can be adopted, we need further laboratory and human studies demonstrating and quantitating the benefit in terms of increased survival without significant neurologic sequelae, while ensuring rescuer, patient, and bystander safety.
